# Systemic delivery of a DUX4-targeting antisense oligonucleotide to treat facioscapulohumeral muscular dystrophy

**DOI:** 10.1016/j.omtn.2021.09.010

**Published:** 2021-09-27

**Authors:** Linde F. Bouwman, Bianca den Hamer, Anita van den Heuvel, Marnix Franken, Michaela Jackson, Chrissa A. Dwyer, Stephen J. Tapscott, Frank Rigo, Silvère M. van der Maarel, Jessica C. de Greef

**Affiliations:** 1 Department of Human Genetics, Leiden University Medical Center, Albinusdreef 2, 2333 ZA Leiden, the Netherlands; 2 Ionis Pharmaceuticals Inc., 2855 Gazelle Court, Carlsbad, CA 92010, USA; 3 Human Biology Division, Fred Hutchinson Cancer Research Center, Seattle, WA 98109, USA; 4 Department of Neurology, University of Washington, Seattle, WA 98105, USA

**Keywords:** facioscapulohumeral muscular dystrophy, DUX4, therapy, antisense oligonucleotide, ACTA1-MCM, FLExDUX4 mouse model

## Abstract

Facioscapulohumeral muscular dystrophy (FSHD) is one of the most prevalent skeletal muscle dystrophies. Skeletal muscle pathology in individuals with FSHD is caused by inappropriate expression of the transcription factor DUX4, which activates different myotoxic pathways. At the moment there is no molecular therapy that can delay or prevent skeletal muscle wasting in FSHD. In this study, a systemically delivered antisense oligonucleotide (ASO) targeting the DUX4 transcript was tested *in vivo* in ACTA1-MCM;FLExDUX4 mice that express DUX4 in skeletal muscles. We show that the DUX4 ASO was well tolerated and repressed the DUX4 transcript, DUX4 protein, and mouse *DUX4* target gene expression in skeletal muscles. In addition, the DUX4 ASO alleviated the severity of skeletal muscle pathology and partially prevented the dysregulation of inflammatory and extracellular matrix genes. DUX4 ASO-treated ACTA1-MCM;FLExDUX4 mice performed better on a treadmill; however, the hanging grid and four-limb grip strength tests were not improved compared to control ASO-treated ACTA1-MCM;FLExDUX4 mice. This study shows that systemic delivery of ASOs targeting DUX4 is a promising therapeutic strategy for FSHD and strategies that further improve the ASO efficacy in skeletal muscle are warranted.

## Graphical abstract

**Figure:**
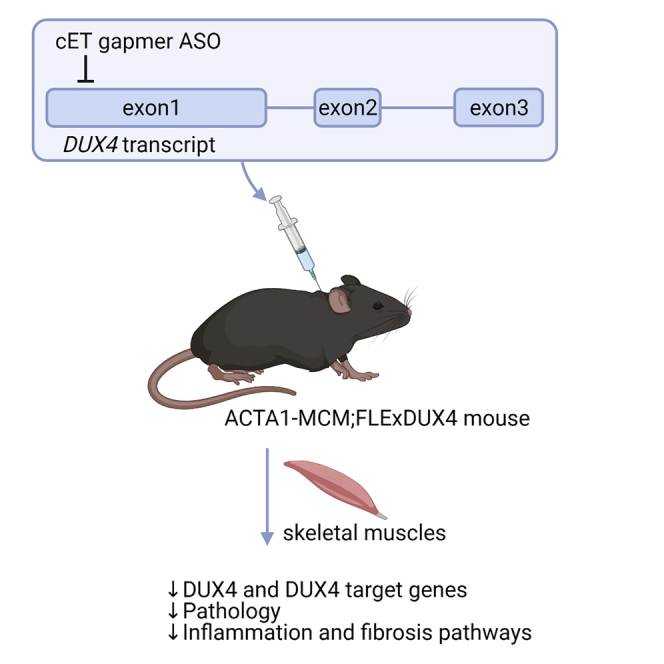

## Teaser

In this study, we show that a systemically delivered antisense oligonucleotide targeting the DUX4 transcript can reduce DUX4 mRNA, DUX4 protein, and DUX4 target gene expression and prevent the dysregulation of inflammatory and extracellular matrix genes in skeletal muscles of ACTA1-MCM;FLExDUX4 mice.

## Introduction

Facioscapulohumeral muscular dystrophy (FSHD) is a progressive skeletal muscle disorder mainly affecting the facial, scapular, and humeral muscles. Individuals with FSHD show clinical heterogeneity, and the disease severity, the age of onset, and which skeletal muscles are affected are highly variable between patients.^1^ Skeletal muscle pathology is caused by epigenetic derepression of the transcription factor double homeobox 4 (*DUX4*).^2^ DUX4 is expressed during the 4-cell stage in human embryos, where it activates the transcription of zygotic genome activation (ZGA) genes.^3^^,^^4^ After early development, the *DUX4* gene is epigenetically repressed in most tissues. Inappropriate expression of DUX4 in skeletal muscles triggers different toxic cascades including, but not limited to, the aberrant expression of germline and ZGA genes, susceptibility to reactive oxygen species, inhibition of nonsense-mediated RNA decay, inhibition of myogenesis, and the induction of apoptotic pathways.^5^, ^6^, ^7^, ^8^, ^9^ The *DUX4* gene is located within the D4Z4 repeat array, a macrosatellite repeat array located on chromosome 4q35. Each D4Z4 unit contains exons 1 and 2 of the *DUX4* gene. *DUX4* is transcribed from the last D4Z4 unit on permissive 4qA alleles that contain exon 3 with a polyadenylation signal (PAS) to stabilize the DUX4 transcript. In the majority of patients (FSHD1), loss of DUX4 repression is caused by a contracted D4Z4 repeat of 1–10 units, whereas non-affected individuals have 8–100 D4Z4 units. An overlap between D4Z4 repeat unit sizes from 8–10 between FSHD and non-affected individuals suggests that more factors are involved in disease penetrance.^2^^,^^10^ In ∼5% of patients (FSHD2), DUX4 derepression is caused by digenic inheritance of a relatively short permissive 4qA allele and mutations in one of the epigenetic D4Z4 repressors SMCHD1, DNMT3B, and LRIF1.^11^, ^12^, ^13^

To date, there is no molecular treatment for patients with FSHD that can stop or slow down disease progression. As the derepression of DUX4 in skeletal muscles causes FSHD, reducing *DUX4* expression is a promising therapeutic strategy that could prevent all toxic downstream effects in the muscle. Several studies have already demonstrated the use of antisense oligonucleotides (ASOs) that target *DUX4* mRNA. 2′-*O*-methyl phosphorothioate ASOs targeting the splice sites or the PAS of the *DUX4* transcript reduced *DUX4* expression, the percentage of DUX4-positive nuclei, and atrophy in FSHD primary myotube cultures.^14^^,^^15^ One of these ASOs was tested as an octa-guanidinium dendrimer conjugated phosphorodiamidate morpholino oligomer (vivo-PMO) in a mouse model with recombinant adeno-associated virus-mediated *DUX4* expression. Intramuscular injections of the ASO downregulated *DUX4* expression in the tibialis anterior muscle.^15^ Two other studies identified an identical PMO targeting the PAS in exon 3 that efficiently repressed *DUX4* and *DUX4* target genes in primary and immortalized FSHD myotubes.^16^^,^^17^ This PAS-targeting PMO was tested *in vivo* by electroporating the PMO into a FSHD muscle xenograft transplanted into the hindlimbs of immunodeficient mice. After the injection of the PMO, the FSHD muscle xenografts showed reduced levels of *DUX4* and *DUX4* target genes.^16^ Lim et al. showed the use of locked nucleic acid (LNA) and 2′-*O*-methoxyethyl (2′-MOE) gapmer ASOs that support the breakdown of *DUX4* mRNA by RNase H.^18^^,^^19^ *In vitro*, different gapmer ASOs, mostly targeting exon 3, reduced the expression of *DUX4* and *DUX4* target genes in immortalized FSHD myotubes. One LNA gapmer ASO and one 2′-MOE gapmer ASO were tested *in vivo* by intramuscular injection in the tibialis anterior muscle of FLExDUX4 (FLExD) mice. FLExD mice carry the DUX4 full-length transgene containing all three exons, two introns, and the PAS on exon 3 in antisense orientation. Because of spontaneous recombination of the *DUX4* transgene, low levels of *DUX4* are expressed.^20^ Both ASOs were able to reduce *DUX4* expression in the injected muscles.^18^^,^^19^ Recently, Lu-Nguyen et al. tested a systemically delivered vivo-PMO targeting exon 3 of DUX4 in FLExD mice that carry an additional ACTA1 skeletal muscle-specific promoter (ACTA1-MCM;FLExD mice) that was induced by Cre-mediated recombination using repeating doses of tamoxifen. The ASO was able to reduce the DUX4 mRNA transcript, DUX4 target gene expression, and pathology in the tibialis anterior muscle.^21^

In this study, an ASO targeting the open reading frame of the DUX4 transcript was tested *in vivo* in ACTA1-MCM;FLExD mice that were not exposed to tamoxifen. Without tamoxifen induction, low levels of *DUX4* are expressed in skeletal muscles, as both the FLExD and the ACTA1-MCM transgenes are leaky. In contrast to FLExD mice, uninduced ACTA1-MCM;FLExD mice show mouse *DUX4* target gene activation and a mild skeletal muscle phenotype.^20^ Different from most *in vivo* studies, the DUX4 ASO was injected subcutaneously for a systemic delivery of the ASO instead of by a local intramuscular injection. We show that the DUX4 ASO reduced *DUX4* mRNA, DUX4 protein, and mouse *DUX4* target gene expression in skeletal muscles of ACTA1-MCM;FLExD mice. In addition, the DUX4 ASO alleviated the severity of skeletal muscle pathology, as shown by a reduction in regenerating fibers, fibrosis, macrophage infiltration, and expression of genes involved in the immune system. In conclusion, we show that systemic delivery of ASOs targeting *DUX4* is a promising therapeutic strategy to treat FSHD.

## Results

### Reduced DUX4 and mouse DUX4 target gene expression in young ACTA1-MCM;FLExD mice receiving a short DUX4 ASO treatment

In this study, a systemically delivered constrained ethyl (cEt) gapmer ASO that targets the open reading frame in exon 1 of the *DUX4* transcript was evaluated. This ASO sequence was the most efficient in repressing DUX4 and human DUX4 target gene expression in a screen performed in FSHD myocytes (data not included in this article). The delivery of the ASO to skeletal muscles was improved by conjugating the ASO to palmitoyl, a fatty acid that facilitates the transport of the ASO from the blood to the skeletal muscles, compared to unconjugated ASOs.^22^ First, to assess whether the DUX4 ASO (DUX4aso) causes severe organ toxicity, wild-type mice were treated for 3 weeks with the DUX4aso (once a week a subcutaneous injection of 100 mg/kg starting at the age of 8 weeks) or injected with phosphate-buffered saline (PBS) as a control (n = 4 per group). During the experiment, body weight and markers for liver toxicity (glutamic oxaloacetic transaminase [GOT] and glutamic pyruvic transaminase [GPT]) were unchanged between the groups (Figures S1A and S1B). The weights of the liver, kidneys, and spleen were recorded after dissection. Only the weight of the liver was slightly increased (Figure S1C); however, we found no evidence of major organ toxicity after exposing the mice to a high dose.

**Figure 1:**
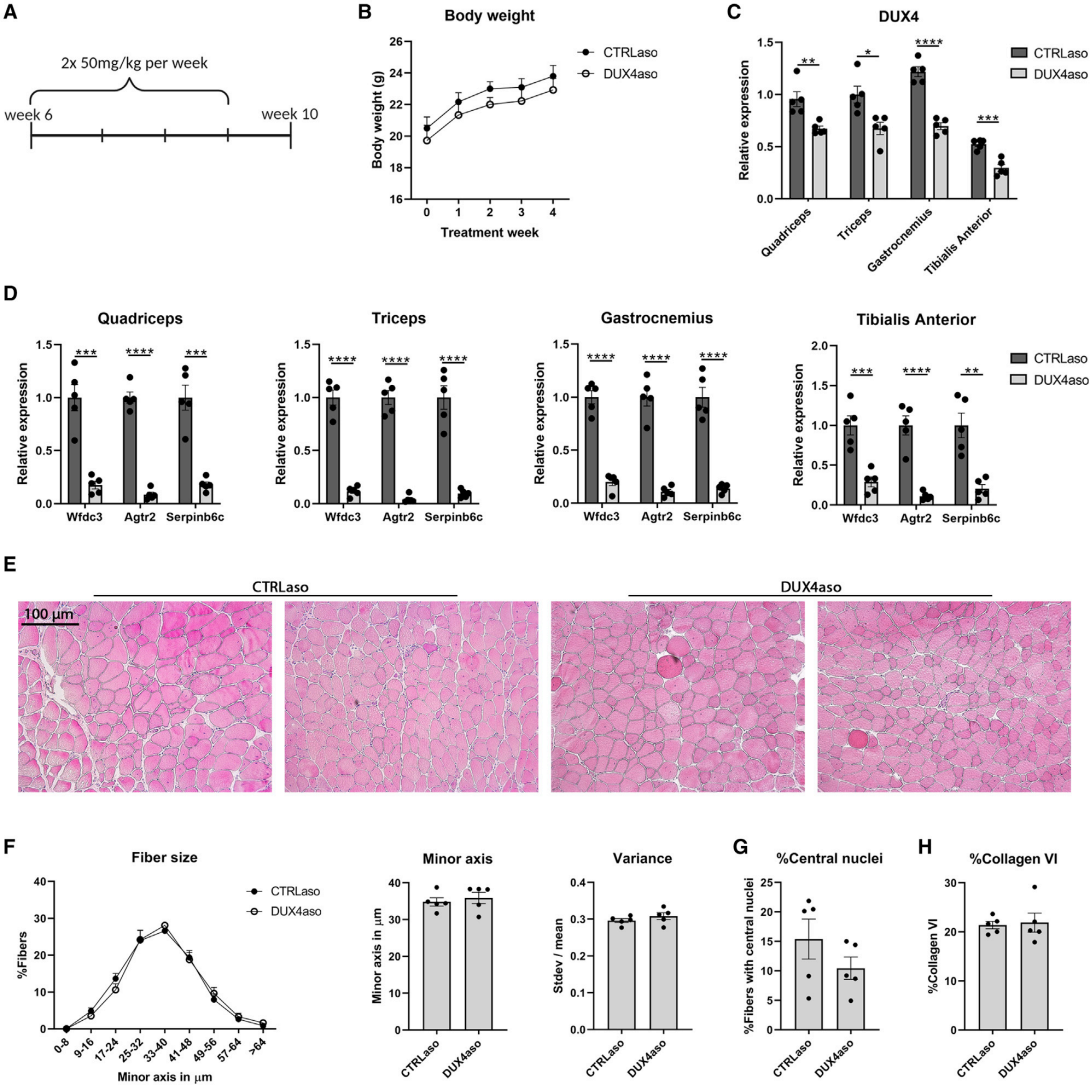
Reduced DUX4 and mouse DUX4 target gene expression in young ACTA1-MCM;FLExD mice receiving a short DUX4 ASO treatment (A) Timeline of the first *in vivo* experiment. From the age of 6 to 9 weeks, ACTA1-MCM;FLExD mice (n = 5 per group) received a dose of 50 mg/kg CTRLaso or DUX4aso twice per week by subcutaneous injection. Mice were euthanized 1 week after the final injection. (B) The body weight in grams during the experiment in both treatment groups. (C) DUX4 expression as measured by qRT-PCR in the quadriceps, triceps, gastrocnemius, and tibialis anterior muscle of DUX4aso- and CTRLaso-treated ACTA1-MCM;FLExD mice. (D) Mouse DUX4 target gene expression (*Wfdc3*, *Agtr2*, and *Serpinb6c*) in four different skeletal muscles as measured by qRT-PCR. (E) Representative H&E stainings (100× magnification) of the quadriceps muscle of DUX4aso- and CTRLaso-treated ACTA1-MCM;FLExD mice at the age of 10 weeks. (F) The fiber size distribution, mean fiber size, and variance (standard deviation of the fiber size divided by the mean fiber size per mouse) in the quadriceps muscle of DUX4aso- and CTRLaso-treated ACTA1-MCM;FLExD mice. (G and H) The percentage of fibers with central nuclei (G) and the percentage of collagen VI-positive staining (H) in the two treatment groups. The amount of collagen VI staining was quantified as the percentage of immunostained area. To determine statistical differences between ACTA1-MCM;FLExD mice treated with CTRLaso (n = 5) or DUX4aso (n = 5), a Student’s t test was used (B–D, F–H). Each dot represents a mouse, and error bars represent the standard error of the mean (SEM). ∗p < 0.05; ∗∗p < 0.01; ∗∗∗p < 0.001; ∗∗∗∗p < 0.0001.

Next, the efficiency of the DUX4aso in repressing DUX4 *in vivo* was tested in hemizygous ACTA1-MCM;FLExD mice. Uninduced hemizygous ACTA1-MCM;FLExD mice develop a mild skeletal muscle pathology from the age of 8–10 weeks that progresses during aging. For our initial experiment, 6-week-old male hemizygous ACTA1-MCM;FLExD mice that had not yet developed a substantial skeletal muscle phenotype received either the DUX4aso or scrambled ASO (CTRLaso) for 3 weeks (twice a week a subcutaneous injection of 50 mg/kg was given), and the mice were sacrificed at the age of 10 weeks (n = 5 per group) (Figure 1A). During the treatment, the DUX4aso did not affect body weight (Figure 1B). In the quadriceps, triceps, gastrocnemius, and tibialis anterior muscle, DUX4 mRNA levels were significantly reduced as measured by qRT-PCR. On average, skeletal muscles showed a 37% reduction in DUX4 transcript levels (Figure 1C). Next, the expression of mouse-specific DUX4 target genes *Wfdc3*, *Agtr2*, and *Serpinb6c* was quantified.^23^^,^^24^ Even though DUX4 was not completely repressed, the DUX4aso could largely prevent the activation of these target genes. In all muscles tested, the target genes were significantly inhibited in DUX4aso-treated mice (Figure 1D). To determine whether the DUX4aso could prevent the onset of skeletal muscle damage, cryosections of the quadriceps muscle were stained with hematoxylin and eosin (H&E) for histochemical analysis. In both groups, skeletal muscle pathology (fibrosis, centrally localized nuclei, inflammation, and necrosis) was still very mild and no overt differences were observed (Figure 1E). To quantify differences in skeletal muscle pathology, the distribution of fiber sizes in the quadriceps muscle was determined. Dystrophic muscles have more degenerating and regenerating fibers, which results in differences in mean fiber sizes and size variability in comparison to non-dystrophic muscles.^25^^,^^26^ The fiber size distribution, mean fiber size, and variance between fibers in the quadriceps muscle were similar in DUX4aso- and CTRLaso-treated mice (Figure 1F). In addition, the percentage of fibers with central nuclei and the percentage of immunostained area for collagen VI as a marker for fibrosis were quantified; however, no changes were found between the two treatment groups (Figures 1G and 1H). In conclusion, a short treatment with this DUX4aso can efficiently repress mouse DUX4 target genes in skeletal muscles; however, at this young age no effect on skeletal muscle pathology was observed.

### Reduced DUX4 and mouse DUX4 target gene expression in adult ACTA1-MCM;FLExD mice receiving a long DUX4 ASO treatment

**Figure 2:**
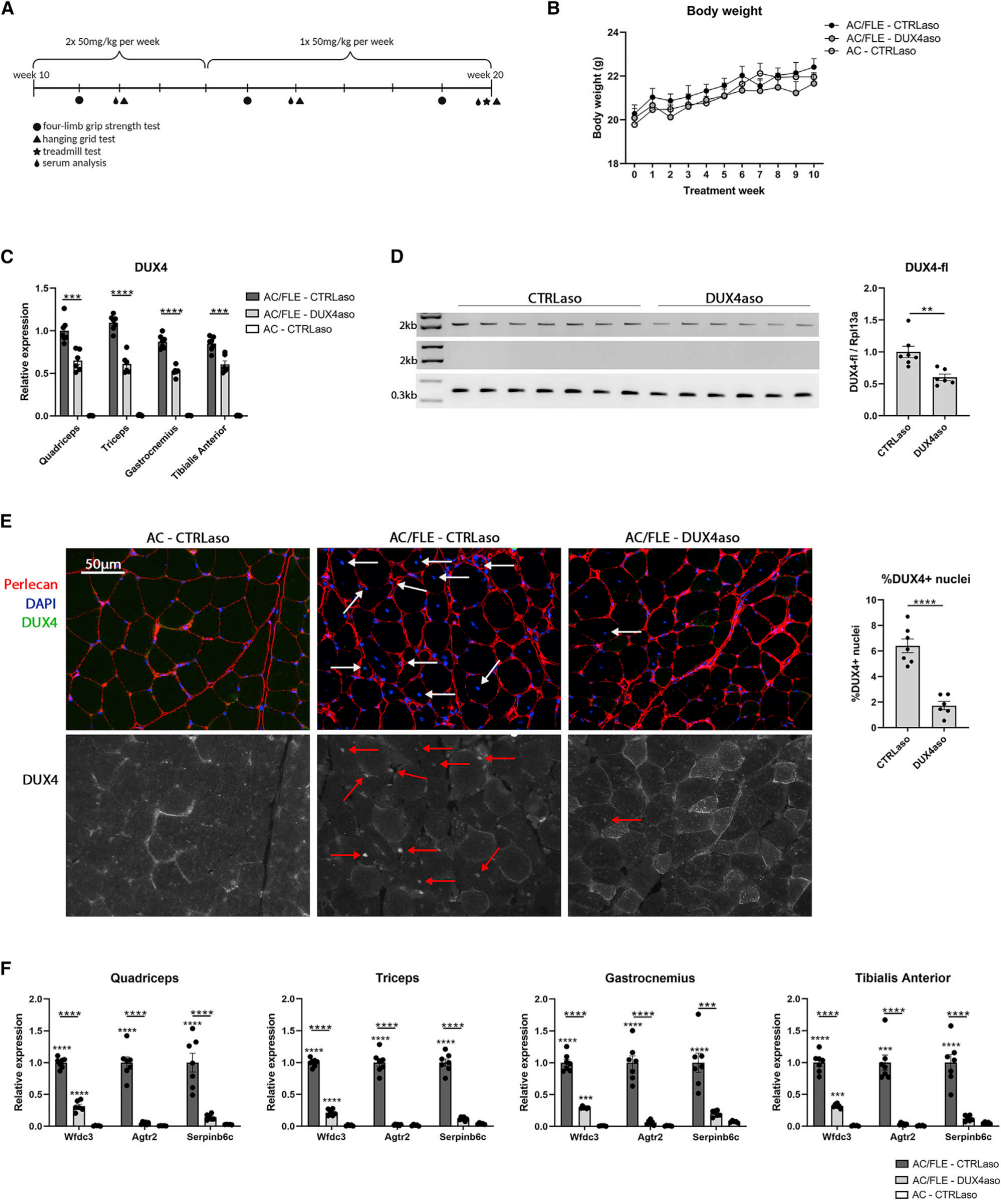
Reduced DUX4 and mouse DUX4 target gene expression in adult ACTA1-MCM;FLExD mice receiving a long DUX4 ASO treatment (A) Timeline of the second *in vivo* experiment. Mice were treated from the age of 10 weeks. In the first 4 weeks, mice received a dose of 50 mg/kg twice per week subcutaneously. In the next 5 weeks, mice received a single dose per week. Mice were euthanized 1 week after the final injection. (B) The body weight in grams in ACTA1-MCM;FLExD mice treated with either the CTRLaso (n = 7) or the DUX4aso (n = 6) and in ACTA1-MCM mice treated with the CTRLaso (n = 5). (C) DUX4 expression in the quadriceps, triceps, gastrocnemius, and tibialis anterior muscle as measured by qRT-PCR. A Student’s t test was used for statistical analysis between CTRLaso- and DUX4aso-treated ACTA1-MCM;FLExD mice. (D) With an endpoint PCR, the DUX4 full-length transcript in the quadriceps muscle was amplified in CTRLaso- and DUX4aso-treated ACTA1-MCM;FLExD mice (first lane). The minus reverse transcriptase control of DUX4 full-length (second lane) did not show any DNA contamination. An endpoint PCR for *Rpl13a* was used as a housekeeping gene (third lane). The amount of full-length DUX4 was quantified by correcting for *Rpl13a* expression. Statistical significance was determined by a Student’s t test. (E) DUX4 immunofluorescence staining on cryosections of the quadriceps muscle and quantification of the percentage of DUX4-expressing nuclei. Arrows indicate DUX4-expressing nuclei. Statistical differences were quantified by a Student’s t test. (F) Expression of mouse DUX4 target genes *Wfdc3*, *Agtr2*, and *Serpinb6c* in skeletal muscles of all three treatment groups. Statistical significance was determined per target gene by a one-way ANOVA. The bar with the large asterisk indicates the statistical differences between DUX4aso- and CTRLaso-treated ACTA1-MCM;FLExD mice. The small asterisk indicates a statistical change in comparison to ACTA1-MCM mice. AC/FLE, ACTA1-MCM;FLExD; AC, ACTA1-MCM. Each dot represents a mouse and the error bars the SEM. ∗p < 0.05; ∗∗p < 0.01; ∗∗∗p < 0.001; ∗∗∗∗p < 0.0001.

To further evaluate the effect of the DUX4aso in ACTA1-MCM;FLExD mice with a skeletal muscle phenotype, a second *in vivo* study was performed. This time, hemizygous ACTA1-MCM;FLExD mice were treated with the CTRLaso (n = 7) or DUX4aso (n = 6) for 10 weeks and were sacrificed at the age of 20 weeks (Figure 2A). At this age, ACTA1-MCM;FLExD mice show a moderate skeletal muscle phenotype in contrast to the first *in vivo* experiment in younger mice. An ACTA1-MCM group receiving a CTRLaso (n = 5) was included to determine whether the DUX4aso can restore muscle weakness, mouse DUX4 target gene expression, and pathology to wild-type levels. Female mice were used for this study as they might suffer from a more severe skeletal muscle phenotype compared to male mice.^27^ In the first 4 weeks, mice received a dose of 50 mg/kg twice per week by subcutaneous injection. In the next 5 weeks, mice received a single dose of 50 mg/kg per week. In addition, functional tests were performed to monitor differences in muscle weakness during the treatment. The body weight between the groups was not different over time (Figure 2B). Similar to the *in vivo* study in wild-type mice, markers for liver toxicity in the serum were low and the weight of several organs was not changed in DUX4aso-treated mice (Figures S2A and S2B).

Similar to the first *in vivo* study, DUX4 mRNA levels were significantly reduced in the quadriceps, triceps, gastrocnemius, and tibialis anterior muscles of DUX4aso-treated ACTA1-MCM;FLExD mice (Figure 2C). The average DUX4 mRNA reduction in skeletal muscles was 37%. A DUX4 full-length PCR with primers spanning exons 1 through 3 showed that the full DUX4 transcript was reduced by 40% on average in the quadriceps muscle of DUX4aso-treated ACTA1-MCM;FLExD mice (Figure 2D). Immunofluorescence stainings for DUX4 were performed on cryosections of the quadriceps muscle to determine whether the reduction in DUX4 mRNA levels also led to a reduction in DUX4 protein. We observed fewer nuclei expressing the DUX4 protein in DUX4aso-treated ACTA1-MCM;FLExD mice in comparison to CTRLaso-treated ACTA1-MCM;FLExD mice. Quantification showed a significant average 73% reduction in the number of DUX4-expressing nuclei in DUX4aso-treated mice (Figure 2E). Mouse DUX4 target genes *Wfdc3*, *Agtr2*, and *Serpinb6c* were measured by qRT-PCR in the quadriceps, triceps, gastrocnemius, and tibialis anterior muscle. In all muscles, the expression of these target genes was significantly reduced in DUX4aso-treated ACTA1-MCM;FLExD mice in comparison to CTRLaso-treated ACTA1-MCM;FLExD mice (Figure 2F). Interestingly, the expression of *Agtr2* and *Serpinb6c* in DUX4aso-treated ACTA1-MCM;FLExD mice was not significantly changed in comparison to ACTA1-MCM mice that do not have the FLExDUX4 transgene, showing that the DUX4aso could reduce the expression of these mouse DUX4 target genes close to levels found in muscles of ACTA1-MCM mice.

### Reduced skeletal muscle pathology in ACTA1-MCM;FLExD mice receiving the DUX4 ASO

**Figure 3:**
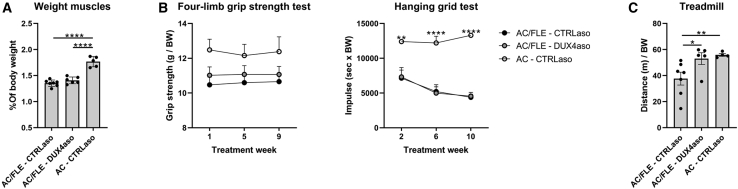
Reduced fatigue but not muscle strength in ACTA1-MCM;FLExD mice receiving the DUX4 ASO (A) The sum of the weight of the quadriceps, triceps, gastrocnemius, and tibialis anterior muscles of ACTA1-MCM;FLExD mice treated with DUX4aso (n = 6) or CTRLaso (n = 7) and ACTA1-MCM mice treated with CTRLaso (n = 5). The muscle weight was corrected for body weight, and statistical significance was measured with a one-way ANOVA. (B) A four-limb grip strength test and a hanging grid test were performed at different time points during the experiment. No statistical differences by two-way ANOVA were measured between the two treatments. (C) A treadmill test with an endpoint of 1,250 m was performed at the end of the treatment. One DUX4aso-treated ACTA1-MCM;FLExD mouse and one CTRLaso-treated ACTA1-MCM mouse were removed from analysis, as they refused to run. Statistical significance was tested by a log rank test (Mantel-Cox). Each dot represents a mouse and the error bars the SEM. BW, body weight in grams; AC/FLE, ACTA1-MCM;FLExD; AC, ACTA1-MCM. ∗p < 0.05; ∗∗p < 0.01; ∗∗∗p < 0.001; ∗∗∗∗p < 0.0001.

To evaluate the effect of the DUX4aso on muscle weakness and muscle pathology, several functional tests and quantifications on muscle sections were performed. The quadriceps, triceps, gastrocnemius, and tibialis anterior muscle were weighed after dissection. The ACTA1-MCM;FLExD mice treated with the CTRLaso showed a reduction in total muscle weight compared to ACTA1-MCM mice (Figure 3A). The DUX4aso could not prevent the loss of muscle mass. To measure differences in muscle strength, a four-limb grip strength test and a hanging grid test were performed at multiple time points (Figure 3B). The four-limb grip strength test did not show statistical differences between the three groups. ACTA1-MCM;FLExD mice treated with the CTRLaso or the DUX4aso had a lower maximum hanging time compared to ACTA1-MCM mice. There were no statistical differences between DUX4aso- and CTRLaso-treated ACTA1-MCM;FLExD mice. To test fatigue in mice, in the last week of the treatment a treadmill test with a maximum of 1,250 m was performed. All ACTA1-MCM mice reached 1,250 m (4 out of 4), whereas none of the ACTA1-MCM;FLExD mice receiving the CTRLaso (0 out of 7) reached 1,250 m. Most ACTA1-MCM;FLExD mice treated with the DUX4aso (3 out of 5) were able to run for 1,250 m. On average, ACTA1-MCM;FLExD mice treated with the DUX4aso performed better than ACTA1-MCM;FLExD mice treated with the CTRLaso (Figure 3C), suggesting that the treatment did not improve muscle strength but might reduce fatigue.

**Figure 4:**
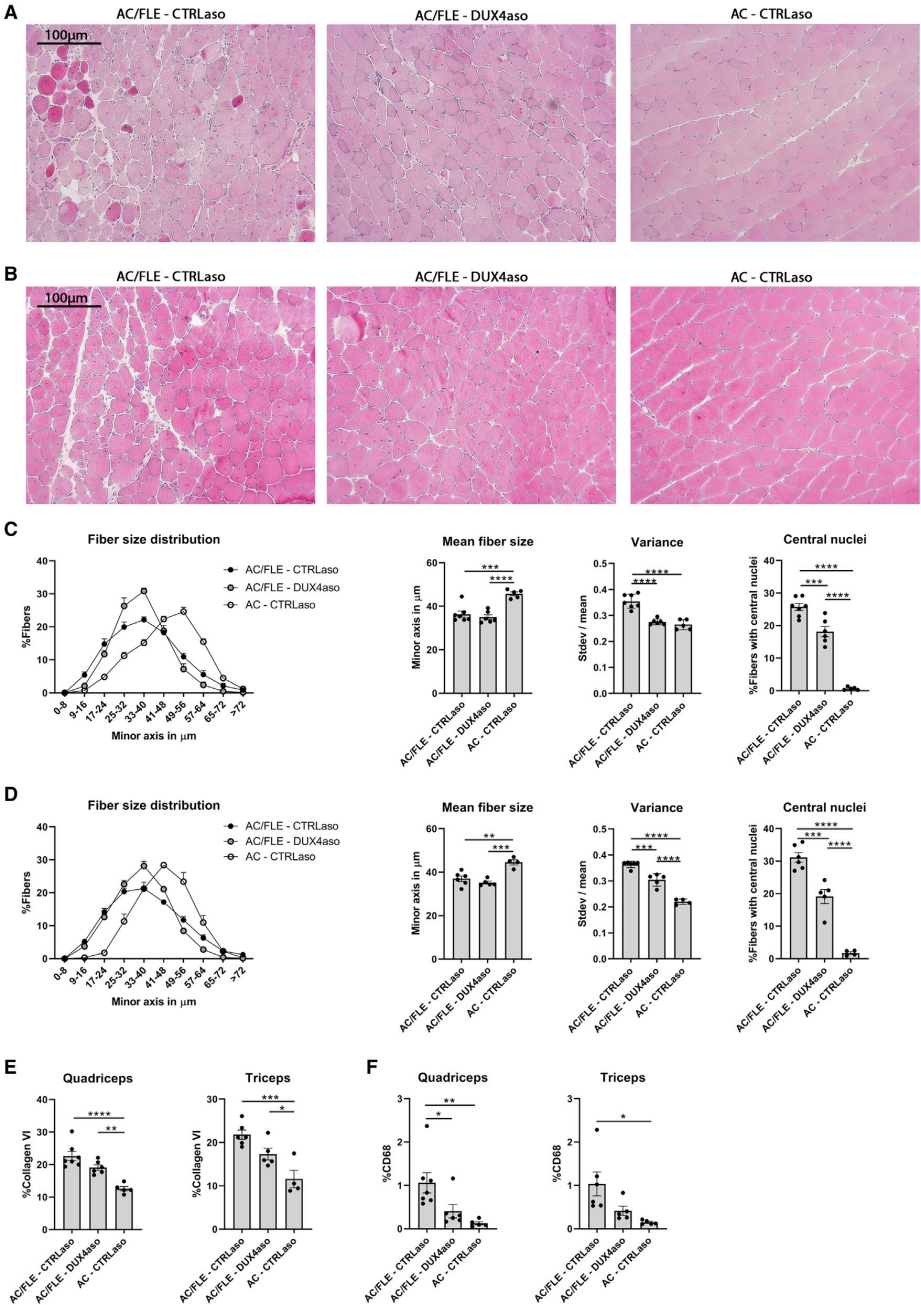
Reduced skeletal muscle pathology in ACTA1-MCM;FLExD mice receiving the DUX4 ASO (A and B) Representative H&E stainings (100× magnification) of the quadriceps (A) and triceps (B) muscles of CTRLaso-treated ACTA1-MCM;FLExD (n = 7), DUX4aso-treated ACTA1-MCM;FLExD (n = 6), and CTRLaso-treated ACTA1-MCM mice (n = 5) at the age of 20 weeks. (C and D) Fiber size distribution, average fiber size, fiber size variance (standard deviation divided by the mean per mouse), and percentage of fibers with central nuclei in the quadriceps muscle (C) and triceps muscle (D) of all three treatment groups. A one-way ANOVA was used for statistical analysis. (E and F) The amount of collagen VI (E) and CD68 (F) staining calculated as percentage of immunostained area in the quadriceps and triceps muscle in all three treatment groups. A one-way ANOVA was used for statistical analysis. AC/FLE, ACTA1-MCM;FLExD; AC, ACTA1-MCM. Each dot represents a mouse and the error bars the SEM. ∗p < 0.05; ∗∗p < 0.01; ∗∗∗p < 0.001; ∗∗∗∗p < 0.0001.

To visualize skeletal muscle pathology, H&E stainings were made of the quadriceps and triceps muscle. Overall, it appeared that skeletal muscle pathology was reduced in the DUX4aso-treated ACTA1-MCM;FLExD mice. For example, fewer mononuclear cell infiltrates and centrally localized nuclei in myofibers were observed (Figures 4A and 4B; Figures S3A and S3B). Next, fiber size distribution, mean fiber size, and variance between fibers were quantified on immunofluorescence stainings of collagen VI in the quadriceps (Figure 4C) and triceps (Figure 4D) muscles. In both muscles, the average fiber size was significantly smaller in ACTA1-MCM;FLExD mice in comparison to ACTA1-MCM mice. No statistical difference in mean fiber size was found between the CTRLaso- and DUX4aso-treated ACTA1-MCM;FLExD mice. Interestingly, in both muscles the DUX4aso reduced the variance in fiber sizes in comparison to the ACTA1-MCM;FLExD mice treated with the CTRLaso (Figures 4C and 4D), which suggests a reduction in regenerating and degenerating muscle fibers. Next, the number of muscle fibers with central nuclei was quantified for each mouse. In ACTA1-MCM mice, the percentage of muscle fibers with central nuclei was low (Figures 4C and 4D). In the quadriceps and triceps muscle of CTRLaso-treated ACTA1-MCM;FLExD mice, the average percentage of fibers with central nuclei was >20%. In both muscles of DUX4aso-treated ACTA1-MCM;FLExD mice we found a significant reduction in the number of fibers with central nuclei, signifying a reduction in regenerating muscle fibers. To verify this result, we performed a staining for Myosin Heavy Chain-embryonic; however, the numbers of Myosin Heavy Chain-embryonic-positive fibers were low in all mice (data not shown). Next, in both muscles the percentage of immunostained area for collagen VI was quantified as a marker for fibrosis. ACTA1-MCM;FLExD mice showed an increased percentage of collagen VI staining in comparison to ACTA1-MCM mice (Figure 4E; Figure S3C). The DUX4aso slightly reduced the collagen VI deposition in both muscles; however, this was not significant. Finally, the percentage of CD68 positivity, a marker for macrophages, was determined (Figure 4F; Figure S3D). In both muscles, a reduction in CD68 positivity was observed in DUX4aso-treated ACTA1-MCM;FLExD mice. In conclusion, the DUX4aso reduced but did not halt skeletal muscle pathology in ACTA1-MCM;FLExD mice.

### The DUX4 ASO reduced DUX4-induced gene expression and biological processes in ACTA1-MCM;FLExD mice

**Figure 5:**
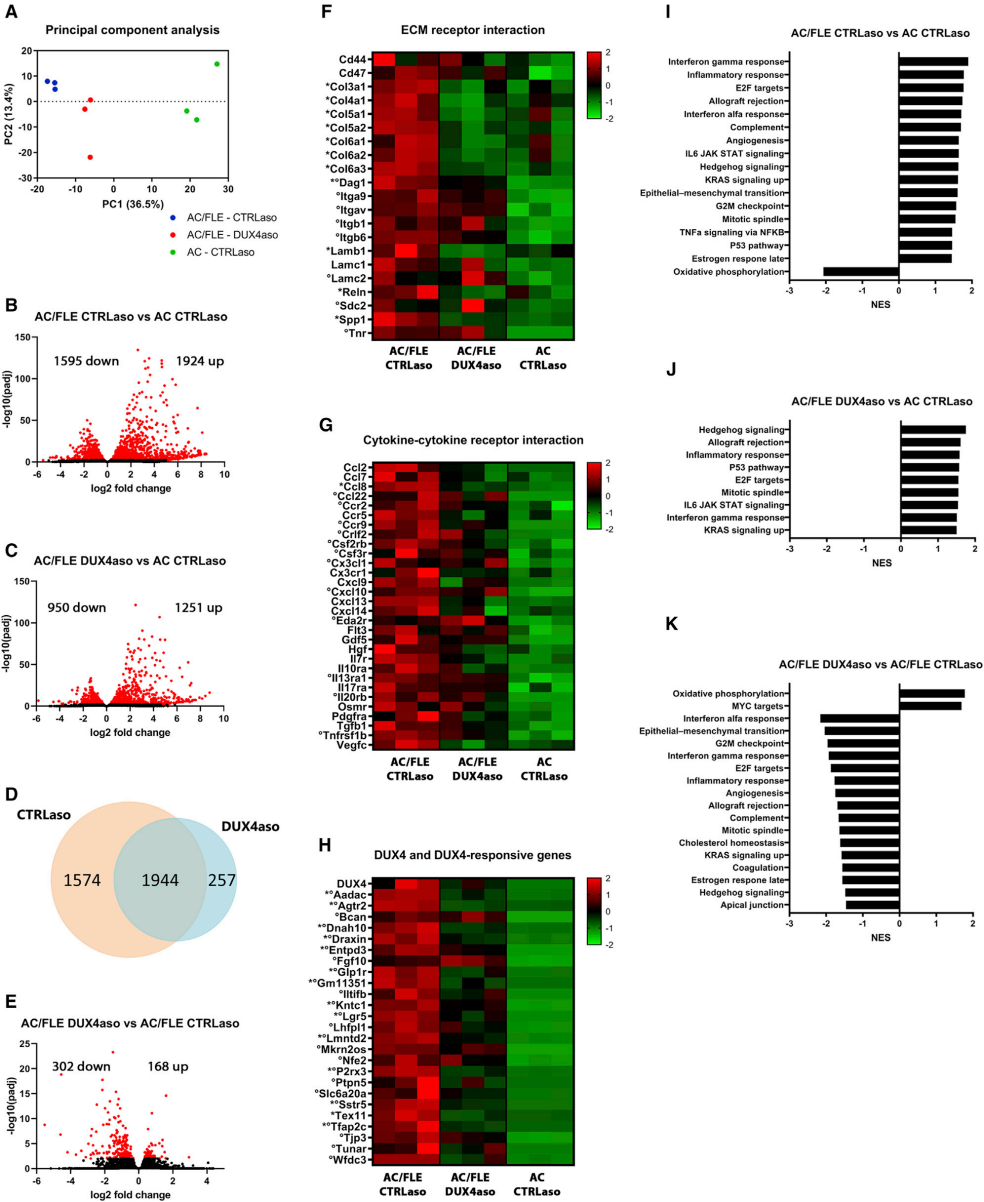
The DUX4 ASO reduced DUX4-induced gene expression and biological processes in ACTA1-MCM;FLExD mice (A) PCA analysis showed that the biological replicates cluster together. ACTA1-MCM mice are further separated from ACTA1-MCM;FLExD mice. (B and C) Volcano plot representations of differential expression analysis of genes in CTRLaso-treated ACTA1-MCM;FLExD (n = 3) mice compared to ACTA1-MCM mice (n = 3) (B) and in DUX4aso-treated ACTA1-MCM;FLExD mice (n = 3) compared to ACTA1-MCM mice (C). Red dots represent differentially expressed genes (adjusted p value < 0.05). (D) Venn diagram representing the overlap between genes differentially expressed in CTRLaso- and DUX4aso-treated ACTA1-MCM;FLExD mice compared to ACTA1-MCM mice. (E) Volcano plot depicting the differential expression results between CTRLaso- and DUX4aso-treated ACTA1-MCM;FLExD mice. Red dots represent differentially expressed genes with an adjusted p value < 0.05. (F–H) Heatmaps showing significantly upregulated genes in CTRLaso-treated ACTA1-MCM;FLExD mice from the KEGG_ECM_RECEPTOR_INTERACTION list (F) and from the KEGG_CYTOKINE_CYTOKINE_RECEPTOR_INTERACTION list (G) and DUX4 transgene and the top 25 mouse DUX4-responsive genes (H). For all three heatmaps, on the scale the *Z* score calculated with the normalized gene counts is depicted. Genes with an asterisk are differentially expressed between CTRLaso- and DUX4aso-treated ACTA1-MCM;FLExD mice. The dots indicate significantly enhanced genes in DUX4aso-treated ACTA1-MCM;FLExD mice compared to ACTA1-MCM mice. (I–K) Gene set enrichment analysis results using the hallmark gene lists in all three comparisons. Bar graphs represent the normalized enrichment score (NES) of significantly enhanced or downregulated biological processes (adjusted p value < 0.05). AC/FLE, ACTA1-MCM;FLExD; AC, ACTA1-MCM.

Sequencing of poly(A)-containing RNA transcripts isolated from the quadriceps muscle from CTRLaso-treated ACTA1-MCM;FLExD mice, DUX4aso-treated ACTA1-MCM;FLExD mice, and ACTA1-MCM mice treated with the CTRLaso was performed to determine whether the treatment can reduce DUX4-induced gene expression and pathways in mice. Principal component analysis (PCA) showed that biological replicates clustered together in the PCA plot (Figure 5A). The ACTA1-MCM mice showed a higher dispersion from the ACTA1-MCM;FLExD mice. In total, CTRLaso-treated ACTA1-MCM;FLExD mice showed 3,519 differentially expressed genes (1,924 up, 1,595 down; p value < 0.05) in comparison to ACTA1-MCM mice (Figure 5B; Data S1). Jones et al. previously reported 855 differentially expressed genes in the gastrocnemius muscles of 13-week-old ACTA1-MCM;FLExD mice (mild model).^27^ Similarly, most of these genes were differentially expressed in CTRLaso-treated ACTA1-MCM;FLExD mice in our analysis; however, we detected more differentially expressed genes (Figure S4). This might be explained by differences in age, different muscles, and differences in data analysis. In total, DUX4aso-treated ACTA1-MCM;FLExD mice showed fewer differentially expressed genes compared to ACTA1-MCM mice (2,201 genes in total; 1,251 up and 950 down) (Figure 5C; Data S1). Differentially expressed genes in the DUX4aso-treated ACTA1-MCM;FLExD mice largely overlapped with the genes that we found differentially expressed in CTRLaso-treated ACTA1-MCM;FLExD mice (1,944 genes; Figure 5D). The DUX4aso did not restore the transcription of these genes to levels found in ACTA1-MCM mice. However, CTRLaso-treated ACTA1-MCM;FLExD mice showed 1,574 other differentially expressed genes compared to ACTA1-MCM mice that were not significantly changed in DUX4aso-treated ACTA1-MCM;FLExD mice compared to ACTA1-MCM mice (Figure 5D), demonstrating that the DUX4aso can partially restore DUX4-induced gene transcription.

DUX4aso-treated ACTA1-MCM;FLExD mice showed 470 differentially expressed genes compared to CTRLaso-treated ACTA1-MCM;FLExD mice (168 genes up, 302 down) (Figure 5E; Data S1). In the list with significantly downregulated genes (Data S1), numerous collagens and other extracellular matrix (ECM) genes were found. A heatmap containing significantly upregulated genes in CTRLaso-treated ACTA1-MCM;FLExD mice compared to ACTA1-MCM mice from the KEGG_ECM_RECEPTOR_INTERACTION lists is depicted in Figure 5F. Eleven out of 21 genes showed a significant reduced expression in DUX4aso-treated ACTA1-MCM;FLExD mice compared to CTRLaso-treated ACTA1-MCM;FLExD mice (indicated with an asterisk). Only 8 out of 21 genes still showed a significant upregulation compared to ACTA1-MCM mice (indicated with a dot), showing that the DUX4aso can reduce the expression of several ECM genes to levels found in ACTA1-MCM mice. This is in line with the quantification of the collagen VI staining (Figure 4E), which showed that the DUX4aso might reduce muscle fibrosis.

Next, we looked at the expression of genes involved in the immune system, as numerous immune genes are upregulated in ACTA1-MCM;FLExD mice.^27^ The second heatmap (Figure 5G) shows the expression of significantly upregulated genes from the KEGG_CYTOKINE_CYTOKINE_RECEPTOR_INTERACTION list in ACTA1-MCM;FLExD mice. In general, the expression of immune genes was lower in DUX4aso-treated ACTA1-MCM;FLExD mice compared to CTRLaso-treated ACTA1-MCM;FLExD mice, which is in line with the reduction in CD68 positivity in skeletal muscles (Figure 4F). However, only Ccl8 showed a significant downregulation. The overall expression levels of immune genes were not restored to levels found in ACTA1-MCM mice, and 12 out of 30 genes were still significantly enhanced in DUX4aso-treated ACTA1-MCM;FLExD mice compared to ACTA1-MCM mice (significantly enhanced genes in DUX4aso-treated mice compared to ACTA1-MCM mice are depicted with a dot).

Next, we looked at the expression of the DUX4 transgene and previously identified murine DUX4-responsive genes obtained from overexpressing DUX4 in C2C12 cells.^23^ DUX4 and the top 25 genes that showed the highest fold change in ACTA1-MCM;FLExD mice compared to ACTA1-MCM mice are depicted in Figure 5H. CTRLaso-treated ACTA1-MCM;FLExD mice showed a significant upregulated expression of DUX4 compared to ACTA1-MCM mice (Data S1). DUX4aso-treated ACTA1-MCM;FLExD mice did not show a significant difference compared to CTRLaso-treated ACTA1-MCM;FLExD or ACTA1-MCM mice; however, the number of reads for the DUX4 transgene in all mice was low or absent. Fourteen out of the 25 murine DUX4-responsive genes were significantly downregulated in DUX4aso-treated mice compared to CTRLaso-treated ACTA1-MCM;FLExD mice (indicated with an asterisk). For most genes, the expression levels were not completely repressed to levels detected in ACTA1-MCM mice, as 24 out of 25 genes were still significantly upregulated (indicated with a dot).

We finally performed gene set enrichment analysis (GSEA) using the hallmark gene sets. Human FSHD muscle biopsies can be distinguished from control biopsies by the expression of genes involved in inflammation, ECM genes (fibrosis), and genes involved in the cell cycle and proliferation.^28^ ACTA1-MCM;FLExD mice showed 16 upregulated biological processes compared to ACTA1-MCM mice (Figure 5I). Although DUX4 activates different target genes in muscles of mice compared to human muscle biopsies, we found that hallmark gene sets involved in inflammation (for example, “Inflammatory response” and “Complement”), ECM (epithelial-mesenchymal transition), and cell cycle (for example, “E2F targets” and “G2M checkpoint”) were enriched in ACTA1-MCM;FLExD mice as well. DUX4aso-treated ACTA1-MCM;FLExD mice showed fewer upregulated processes compared to ACTA1-MCM mice (Figure 5J). Comparing DUX4aso-treated ACTA1-MCM;FLExD mice with CTRLaso-treated ACTA1-MCM;FLExD mice showed that many biological processes, including gene sets involved in inflammation, fibrosis, and cell cycle, were repressed by the DUX4aso (Figure 5K). Overall, the RNA sequencing data show that the DUX4aso reduced toxic pathways induced by DUX4 expression that are found in human FSHD biopsies as well.

## Discussion

FSHD is one of the most prevalent progressive muscular dystrophies. To date, there is no molecular therapy that can halt or slow down skeletal muscle wasting.^1^ As skeletal muscle pathology is caused by derepression of the transcription factor DUX4, inhibiting the DUX4 transcript could halt the activation of all downstream toxic cascades.^2^ In this study, an ASO targeting the open reading frame of the DUX4 transcript was tested *in vivo* by subcutaneous injection in the ACTA1-MCM;FLExD mouse model that suffers from a progressive skeletal muscle pathology. We show that the systemic delivery of the DUX4aso reduced *DUX4* mRNA, DUX4 protein, and mouse *DUX4* target gene expression in all tested skeletal muscles (Figures 1 and 2). In addition, the DUX4aso was able to decrease the severity of skeletal muscle pathology (Figure 4) in ACTA1-MCM;FLExD mice and partially inhibited DUX4-induced gene expression (Figure 5).

Several ASOs have shown beneficial results in patients with neuromuscular disorders.^29^, ^30^, ^31^ For FSHD, no ASOs have yet been tested in patients; different studies, however, have shown that ASOs were efficient in reducing *DUX4* and *DUX4* target genes in FSHD myocytes and in FSHD mice.^15^, ^16^, ^17^, ^18^, ^19^^,^^21^ A major advantage of our systemic approach compared to most other *in vivo* studies in FSHD mice is that all tested skeletal muscles were targeted by the ASO instead of one muscle or a part of the muscle. Next, this is the first ASO with cEt chemistry that has been tested for FSHD. In previous studies PMO, 2′-MOE, and LNA chemistries have been used. *In vivo*, PMOs often show poor uptake by target tissues and fast clearance from the circulation. This can be improved by using 2′-MOE or LNA chemistries. In general, LNA gapmers show a stronger affinity to the target RNA, higher RNase H-mediated cleavage activity, and reduced degradation by nucleases compared to 2′-MOE gapmers; however, the development of some LNA gapmers has been hampered, as they induced hepatotoxicity.^32^^,^^33^ cEt-modified gapmers show characteristics similar to LNA gapmers, but with reduced toxicity levels.^34^ In our study we did not find evidence of major organ toxicity. All serum markers for liver damage were low. In addition, total body weight and organ weight were not changed in DUX4aso-treated ACTA1-MCM;FLExD mice (Figure 2B; Figure S2). Previously, systemically delivered cEt gapmers showed high target gene reductions in mice with neuromuscular disorders.^35^, ^36^, ^37^ However, a Phase 1/2a study testing a DM1 Protein Kinase gene (DMPK) targeting cEt ASO in subjects with myotonic dystrophy was discontinued because the drug concentration in tissue was not high enough to elicit expected splicing changes (L.N. Mignon et al., 2016, Am. Acad. Neurol., abstract).

Nonetheless, the DUX4aso might target DUX4c and DUXO, as the DUX4aso has complementarity to these genes. In addition, the DUX4aso has partial complementarity to several other genes including DUX1 and DUX5. Previous studies showed that DUX4c is upregulated in FSHD myocytes and that it may disturb myogenesis and facilitate DUX4 toxicity,^38^^,^^39^ although in one FSHD family a proximal deletion at D4Z4 including the DUX4c gene was identified and patients have been diagnosed with FSHD linked to chromosome 10q where no complete DUX4c gene resides, suggesting that DUX4c is dispensable for FSHD pathogenesis.^38^^,^^40^^,^^41^ The DUXO gene may have a function in early development.^42^ We therefore do not expect that reducing DUX4c and DUXO transcript levels in skeletal muscles will cause adverse effects; however, we could not assess this in the current study because DUX4c and DUXO are absent from the mouse genome.

The DUX4aso was able to reduce skeletal muscle pathology in ACTA1-MCM;FLExD mice. Nevertheless, skeletal muscles of DUX4aso-treated ACTA1-MCM;FLExD mice still showed signs of skeletal muscle pathology, including smaller muscle fibers and more centrally located nuclei compared to ACTA1-MCM mice (Figure 4). RNA sequencing still showed quite a number of differentially expressed genes and upregulation of a few hallmark gene sets compared to ACTA1-MCM mice (Figures 5C and 5J). It seems that the DUX4aso can reduce skeletal muscle pathology but cannot restore it to levels found in ACTA1-MCM mice. In addition, the four-limb grip strength test and hanging grid test did not show an improvement in DUX4aso-treated ACTA1-MCM;FLExD mice (Figure 3B). From the beginning of the treatment at 10 weeks of age, the ACTA1-MCM;FLExD mice already presented with muscle weakness compared to ACTA1-MCM mice. The DUX4aso may not be able to restore muscle wasting once it has already been established. Another explanation could be the modest DUX4 transcript reduction in the skeletal muscles of only 37% (Figure 1C, Figure 2C). In contrast, the reduction in DUX4 protein (73% fewer DUX4-expressing nuclei) and in mouse *DUX4* target gene expression was more efficient (Figures 2E and 2F). It is unclear how this modest reduction in DUX4 RNA expression can largely prevent the translation of the DUX4 protein and the activation of mouse *DUX4* target genes. Several explanations may underlie this observation, including the sporadic presence of DUX4-positive myonuclei,^43^ DUX4 protein diffusion to neighboring myonuclei,^44^^,^^45^ the nucleocytoplasmic distribution of DUX4 RNA,^46^ or the ASO might efficiently bind to the DUX4 RNA, blocking its translation, but could not efficiently recruit RNase H. Nevertheless, the residual DUX4 protein in myonuclei of DUX4aso-treated ACTA1-MCM;FLExD mice might still induce skeletal muscle pathology. A more efficient DUX4 knockdown could be achieved by improving the delivery toward skeletal muscles by using a different conjugation of the ASO, for example, by using cell-penetrating peptides.^47^ Next, based on the four-limb grip strength test, the muscle force of ACTA1-MCM;FLExD mice barely declined during the 10-week treatment. This may explain why we did not measure any functional differences, except for fatigue, between the two treatments. To determine the efficiency of the DUX4aso on skeletal muscle pathology and muscle weakness, it may be better to start the treatment (1) before the first symptoms start or (2) at an age where the ACTA1-MCM;FLExD mice show a rapid decline in muscle strength, (3) use repetitive low doses of tamoxifen, (4) treat mice over a longer period of time, or (5) test the ASO in male mice, as they are less severely affected and might be more likely to show a significant improvement.^27^

Taken together, using ACTA1-MCM;FLExD mice that have low levels of DUX4 and a moderate skeletal muscle phenotype, we showed that the systemically delivered DUX4aso is well tolerated and can decrease the *DUX4* transcript, DUX4 protein, and mouse *DUX4* target genes in skeletal muscles. In addition, the DUX4aso was able to reduce several hallmarks of skeletal muscle pathology, including the percentage of myofibers with central nuclei and the expression of different inflammation gene lists. Altogether, this study demonstrates that systemically delivered ASOs targeting DUX4 are promising therapeutic strategies to treat patients with FSHD. Future studies will focus on increasing skeletal muscle specificity of the ASO and gaining more insight into potential off-target effects and organ toxicity.

## Materials and methods

### Mouse husbandry and genotyping

Wild-type mice on a C57BL/6J background that were used for the first toxicity experiment were kept at Ionis Pharmaceuticals. All protocols met ethical standards for animal experimentation and were approved by the Institutional Animal Care and Use Committee of Ionis Pharmaceuticals. Transgenic FLExDUX4 (FLExD) and ACTA1-MCM mice were housed at the animal facility of the Leiden University Medical Center (LUMC). Experiments at the LUMC were carried out according to Dutch law and Leiden University guidelines and were approved by the National and Local Animal Experiments Committees. All mice were housed in individually ventilated cages with a standard 12-h/12-h light/dark cycle. Standard rodent chow and water were available *ad libitum*. FLExD mice were generated and described previously and kindly provided to us by Dr. Jones (University of Nevada, Reno, NV, USA).^20^ The ACTA1-MCM line (ACTA1-MerCreMer, 025750) was purchased from Jackson Labs (Bangor, ME, USA). Hemizygous ACTA1-MCM;FLExD and ACTA1-MCM mice were obtained by cross-breeding hemizygous FLExD mice with hemizygous ACTA1-MCM mice on a C57BL/6J background. All mice were euthanized by cervical dislocation. Genotyping was performed on DNA isolated from the tail. For the detection of the FLExDUX4 transgene, the following primers were used: 5′-CAATACCTTTCTGGGAGTTCTCTGCTGC-3′ and 5′-CTCGTGTAGACAGAGCCTAGACAATTTGTTG-3′. To detect the ACTA1-MCM transgene, the following primers were used: 5′-ATGTCCAATTTACTGACCGTACAC-3′ and 5′-GCCGCATAACCAGTGAAACA-3′.

### ASO treatment of mice

All chemically modified oligonucleotides were synthesized and purified as previously described.^48^ The ASOs are 16 nucleotides in length, wherein the central gap segment comprising ten 2′-deoxyribonucleotides is flanked on the 5′ and 3′ wings by three cEt-modified nucleotides. Internucleotide linkages were phosphorothioate, and all cytosine residues were 5′-methylcytosines. ASOs are conjugated at the 5′ end with palmitate. The control ASO (5′-GGCCAATACGCCGTCA-3′) and the DUX4 ASO (5′-GGCGATGCCCGGGTAC-3′) were dissolved in sterile PBS to a concentration of 10 mg/mL. For the toxicity experiment, wild-type mice were subcutaneously injected with a dose of 100 mg/kg once per week or with an equal volume of PBS. For the other *in vivo* experiments, ACTA1-MCM;FLExD and ACTA1-MCM mice were subcutaneously injected with a dose of 50 mg/kg once or twice per week. For the short *in vivo* experiment in ACTA1-MCM;FLExD mice, male mice were used. Female mice were used for the toxicity experiment in wild-type mice and the second long *in vivo* experiment in ACTA1-MCM;FLExD and ACTA1-MCM mice.

### Functional tests

For multiple time points, the four-limb grip strength test and hanging grid test were performed to measure muscle weakness. For the four-limb grip strength test, the mouse was placed on a flat mesh pull bar attached to an isometric force transducer (Columbus Instruments, Columbus, OH, USA). The mouse was pulled away from the mesh by its tail, and the force was recorded by the force transducer. For each mouse, the test was repeated five times, with 1-min rest in between, within the same session. The mean of the three highest values was used for analysis and corrected for body weight. For the hanging grid test, the mouse was placed on a grid that was inverted, and the hanging time was recorded. The mouse had three attempts to hang onto the grid, unless a maximum hanging time of 600 s was reached. The best hanging time was used for analysis and corrected for body weight. For the treadmill test, mice were exercised on an adjustable variable-speed belt treadmill with a built-in shock grid from OmniPacer (Accuscan Instruments, Columbus, OH, USA). Mice were first acclimatized at a speed of 5 m/min for 5 min at 0° incline. The test was performed with an initial speed of 8 m/min, with speed increasing by 1 m/min every 10 min. Mice were run until exhaustion or to a maximum of 1,250 m. Two mice that refused to run were removed from the analysis. The experimenter was blinded to the genotypes and treatments of individual mice.

### Serum analysis

A small cut in the tail was made, and blood was collected in EDTA-coated Microvettes (Sarstedt, the Netherlands). The Microvette was centrifuged for 10 min at 4°C, and serum was transferred to a new tube. Serum was 1:5 diluted in PBS, and 30 μL of this dilution was used per test. The following test strips were used: Reflotron GPT (alanine aminotransferase [ALT]), Reflotron GOT (aspartate aminotransferase [AST]), and Reflotron ALP (alkaline phosphatase). All samples were analyzed with the Reflotron Sprint device (Roche, Basel, Switzerland).

### qRT-PCR and endpoint PCR

Tissues were first homogenized in Qiazol (QIAGEN, Venlo, the Netherlands). RNA was extracted and purified with the miRNeasy mini kit (QIAGEN, Venlo, the Netherlands) according to the manufacturer’s instructions. RNA was treated with DNase on the column for 30 min at room temperature (RT). The concentration of eluted RNA was measured with the NanoDrop ND-1000 spectrophotometer (Thermo Fisher Scientific, Bleiswijk, the Netherlands). cDNA was synthesized from 3 μg of RNA with the RevertAid H Minus First Strand cDNA Synthesis Kit using oligo(dT) primers (Thermo Fisher Scientific, Bleiswijk, the Netherlands). Gene expression levels were determined by qRT-PCR with the CFX96 system (Bio-Rad, Veenendaal, the Netherlands) using iQ SYBR Green Supermix (Bio-Rad, Veenendaal, the Netherlands) and 0.5 pM forward and reverse primer (Table 1). The following qRT-PCR program was used: 95°C for 3 min, 40 cycles of 10 s at 95°C and a melting temperature of 60°C for 30 s, followed by a melting curve analysis from 65°C to 95°C (temperature increments of 0.5°C). Quantification cycle (Cq) values were obtained from Bio-Rad CFX Manager version 3.1 software (Bio-Rad, Veenendaal, the Netherlands) and were normalized for the housekeeping genes *Rpl13a* and *Gapdh*. For the DUX4 full-length RT-PCR, an endpoint PCR was performed with LA Taq DNA polymerase and LA buffer I (Takara Bio Europe, Saint-Germain-en-Laye, France). Rpl13a was used as a loading control. PCR products were visualized on a 2% (DUX4 full length) or 1% (Rpl13a) agarose gel. Quantification of the PCR product was performed with ImageJ (National Institutes of Health, Bethesda, MD, USA).

**Table 1:** List of qRT-PCR and endpoint PCR primers

Gene	Forward	Reverse
Agtr2	5′-CGGGAGCTGAGTAAGCTGAT-3′	5′-GACGGCTGCTGGTAATGTTT-3′
DUX4	5′-TTTTTTTTTTTTTTTTTCTATAGGATCCACAGG-3′	5′-CTTCCGTGAAATTCTGGCTGAATG-3′
DUX4 full-length (endpoint PCR)	5′-cgaggacggcgacggagac-3′	5′-gatccacagggagggggcatttta-3′
Gapdh	5′-TCCATGACAACTTTGGCATTG-3′	5′-TCACGCCACAGCTTTCCA-3′
Rpl13a	5′-TGCTGCTCTCAAGGTTGTTC-3′	5′-TTCTCCTCCAGAGTGGCTGT-3′
Rpl13a (endpoint PCR)	5′-GGAAGCGGATGAATACCAAC-3′	5′-TGCTTCTTCTTCCGATAGTGC-3′
Serpinb6c	5′-CAAAGAGGACACCAGGGAGA-3′	5′-AGCTCATTGCCAACATAGGA-3′
Wfdc3	5′-CTTCCATGTCAGGAGCTGTG-3′	5′-ACCAGGATTCTGGGACATTG-3′

### Histology

Skeletal muscles were dissected from euthanized mice, embedded in O.C.T. Compound (Tissue-Tek; Sakura Finetek, Torrance, CA, USA), rapidly frozen in cooled isopentane, and, when frozen, transferred to liquid nitrogen. Cryosections of 7 μm were made with a cryotome. To visualize muscle pathology, cryosections were first stained with hematoxylin for 5 min, followed by eosin staining for 1 min. Cryosections were dehydrated by increasing ethanol concentrations (from 50% to 100%) and finished by incubating the slides in xylene for 5 min. Slides were enclosed in Entellan (Merck, Amsterdam, the Netherlands). Pictures were made with light microscopy (Leica Microsystems, Amsterdam, the Netherlands).

### Muscle fiber size, central nuclei, collagen VI, and CD68 quantification

Cryosections were fixed in 4% paraformaldehyde for 10 min. Sections were blocked in 10% normal donkey serum (Abcam, Cambridge, UK) for 30 min. Blocked sections were incubated with 1:150 diluted rabbit anti-collagen type VI antibody (70R-CR009X; Bio-Connect, Huissen, the Netherlands) and 1:100 rat anti-CD68 antibody (BioLegend, London, UK) in PBS-0.1% BSA for 1 h at RT. Thereafter, sections were incubated for 30 min at RT with 1:500 diluted anti-rabbit immunoglobulin G (IgG) H&L Alexa Fluor 488 and anti-rat IgG H&L Alexa Fluor 594 (Thermo Fisher Scientific, Bleiswijk, the Netherlands) in PBS-0.1% BSA. Nuclei were stained with DAPI (Thermo Fisher Scientific, Bleiswijk, the Netherlands) for 15 min at RT. Sections were enclosed in Aqua-Poly/Mount (Polysciences, Hirschberg, Germany), and pictures were made with the Leica DM5500 microscope (Leica Microsystems, Amsterdam, the Netherlands) with a 100× magnification. For the quantification of the muscle fiber sizes (at least 1,000 fibers per mouse), five randomly taken pictures were analyzed per mouse with BZ-X Analyzer software (Keyence, Osaka, Japan). The number of central nuclei was counted by hand by two blinded persons. The amount of collagen VI/CD68 staining was quantified with ImageJ software as the percentage of immunostained area on at least five randomly taken pictures per mouse (National Institutes of Health, Bethesda, MD, USA).

### DUX4 immunofluorescence staining

Cryosections were fixed in 4% paraformaldehyde for 10 min. Sections were blocked in blocking solution (1% normal donkey serum [Abcam, Cambridge, UK], 1% BSA) for 30 min at RT and incubated overnight at 4°C in the following primary antibody mix: 1:100 diluted rabbit C-terminal anti-DUX4 E5-5 (Abcam, Cambridge, UK) and 1:200 diluted rat anti-Perlecan A7L6 (Thermo Fisher Scientific, Bleiswijk, the Netherlands) in blocking solution. The sections were stained with the following secondary antibody mix: 1:500 diluted anti-rat IgG H&L Alexa Fluor 594 (Abcam, Cambridge, UK) and 1:500 diluted anti-rabbit H&L Alexa Fluor 488 (Thermo Fisher Scientific, Bleiswijk, the Netherlands) for 1 h at RT in blocking buffer. DAPI (Thermo Fisher Scientific, Bleiswijk, the Netherlands) was used to stain nuclei. Sections were enclosed in Aqua-Poly/Mount (Polysciences, Hirschberg, Germany), and pictures were randomly made with the Leica DM5500 microscope (Leica Microsystems, Amsterdam, the Netherlands) with a 200× magnification. The number of nuclei was quantified with ImageJ software (National Institutes of Health, Bethesda, MD, USA), and the number of DUX4-positive nuclei was counted by hand by two blinded experimenters.

### Bulk RNA sequencing and analysis

Total RNA quality of the quadriceps RNA samples derived from the long *in vivo* study in ACTA1-MCM;FLExD and ACTA1-MCM mice was analyzed with the Agilent BioAnalyzer RNA Nano 6000 chip (Agilent Technologies, Amstelveen, the Netherlands). All samples used for bulk RNA sequencing had a RNA Integrity Number of ≥7.9. For each group (ACTA1-MCM;FLExD CTRLaso, ACTA1-MCM;FLExD DUX4aso, ACTA1-MCM CTRLaso), the poly(A)-containing transcripts of three samples were sequenced by GenomeScan (Leiden, the Netherlands) with the NovaSeq 6000 PE150 system (Illumina). Reads were trimmed and quality filtered by TrimGalore (v.0.4.5, Cutadapt v.1.16), using default parameters to remove low-quality nucleotides (error rate < 0.05). The reads were mapped to Genome Reference Consortium Mouse Build 38, Gencode release M24, and the FLExDUX4 mRNA sequence with STAR Aligner (v.2.5.1b). PCR duplicates were removed from analysis based on unique molecular identifiers with UMI-tools (v.1.0.1). A gene expression counts table was generated with HTSeq (v.0.9.1, genome annotation vM24). Data were next sequence depth-normalized following the median of ratios method implemented in the DESeq2 R package (v.1.24.0). Genes with an adjusted p value below 0.05 (Benjamini-Hochberg) were considered significant. PCA analysis was performed with the prcomp function using the R stats package. GSEA using the hallmark gene lists was performed with GSEA 4.1.0. software.^49^ Gene lists with an adjusted p value below 0.05 were considered significant. For the heatmaps, the KEGG pathways lists were downloaded from GSEA and the *Z* scores were calculated using normalized gene counts derived with the DESeq2 R package (v.1.24.0). Graphs were made in GraphPad Prism software (version 8; GraphPad Software, La Jolla, CA, USA).

### Statistics

GraphPad Prism software (version 8; GraphPad Software, La Jolla, CA, USA) was used to perform statistical tests. The figure legends describe which statistical test was used per experiment. All error bars represent the standard error of the mean. p values of <0.05 were considered significant. ∗p < 0.05; ∗∗p < 0.01; ∗∗∗p < 0.001; ∗∗∗∗p < 0.0001.

## Supplemental information

Document 1. Figures S1–S4

Data S1. List of genes up- or down-regulated (adjusted p value <0.05) in the quadriceps muscle of ACTA1-MCM;FLExD and ACTA1-MCM mice receiving a long DUX4 ASO treatment

Document 2. Article plus supplemental information
