# Supplementary material for: Systemic delivery of a DUX4-targeting antisense oligonucleotide to treat facioscapulohumeral muscular dystrophy
Source: Mol Ther Nucleic Acids. 2021 Sep 27;26:813–27. doi: 10.1016/j.omtn.2021.09.010 (PMC8526479; doi:10.1016/j.omtn.2021.09.010)
Supplement: Document 1. Figures S1–S4 [file mmc1.pdf]

OMTN, Volume *26*

Supplemental information

Systemic delivery of a DUX4-targeting antisense oligonucleotide to treat facioscapulohumeral muscular dystrophy

Linde F. Bouwman, Bianca den Hamer, Anita van den Heuvel, Marnix Franken, Michaela Jackson, Chrissa A. Dwyer, Stephen J. Tapscott, Frank Rigo, Silvère M. van der Maarel, and Jessica C. de Greef

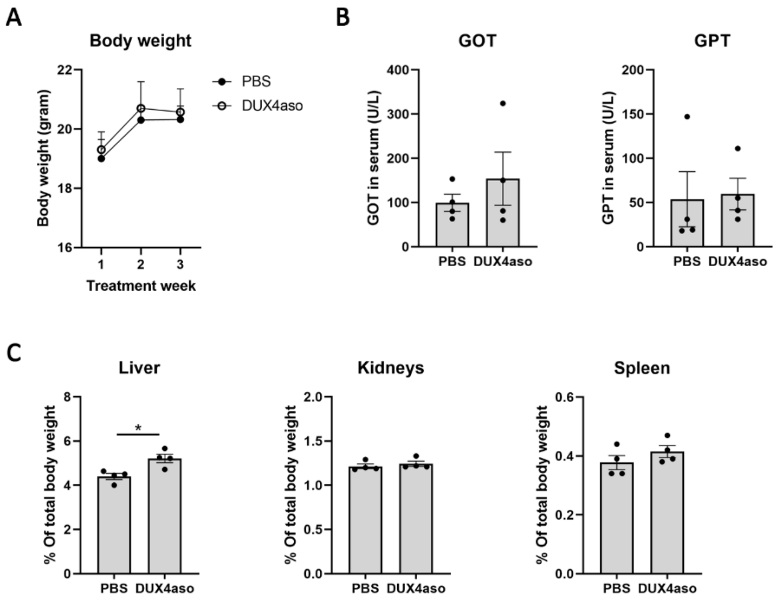

Figure S1: Markers for organ toxicity were not changed in DUX4aso treated wild-type mice compared to PBS-injected mice. (A) The average body weight in PBS or DUX4aso treated wild-type mice during a treatment for three weeks (100 mg/kg). (B) Serum markers for liver toxicity (GOT, GPT) after the final treatment. (C) The weight of the liver, kidneys and spleen corrected for body weight. Statistical analysis was performed using a Student's T-test. Each dot represents a mouse and the error bars the SEM. *P<0.05; **P<0.01; ***P<0.001; ****P<0.0001.

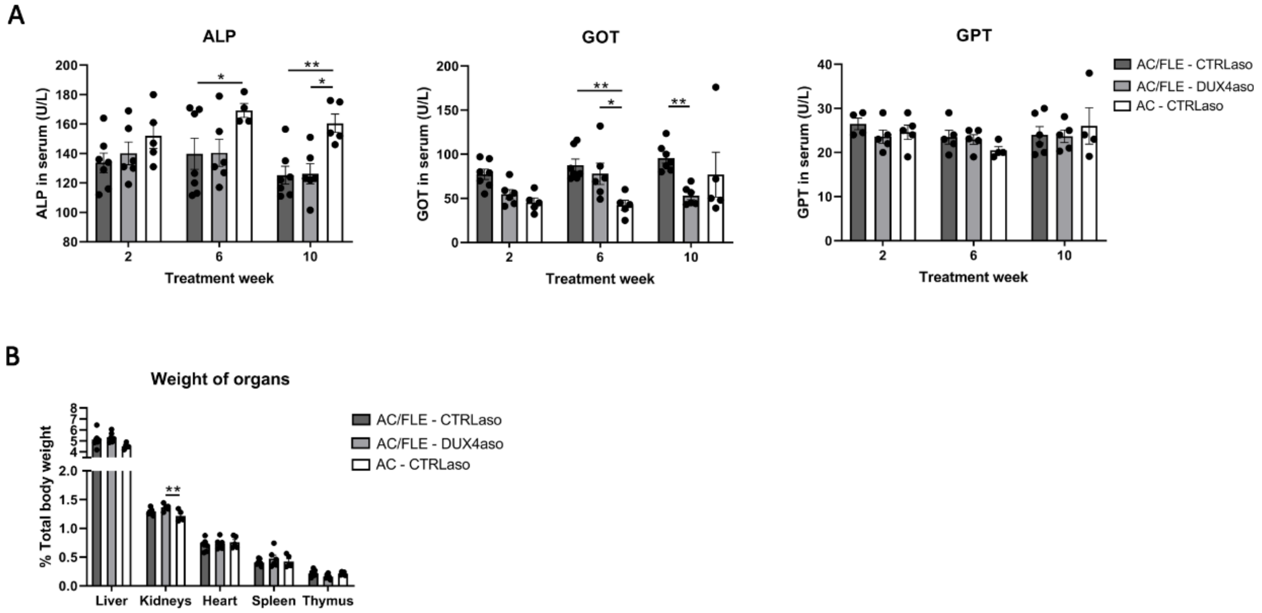

Figure S2: Markers for organ toxicity in DUX4aso treated ACTA1-MCM;FLExD mice were not changed. (A) Serum markers for liver toxicity (ALP, GOT, GPT) were measured during multiple timepoints in CTRLaso ACTA1-MCM;FLExD mice, DUX4aso treated ACTA1-MCM;FLExD mice, and CTRLaso treated ACTA1-MCM mice. Statistical analysis was performed per marker using a one-way ANOVA. (B) The weight of different organs corrected for body weight in all three treatment groups. Statistical changes per organ were measured using a one-way ANOVA. AC/FLE = ACTA1-MCM;FLExD; AC = ACTA1-MCM. Each dot represents a mouse and the error bars the SEM. *P<0.05; **P<0.01; ***P<0.001; ****P<0.0001.

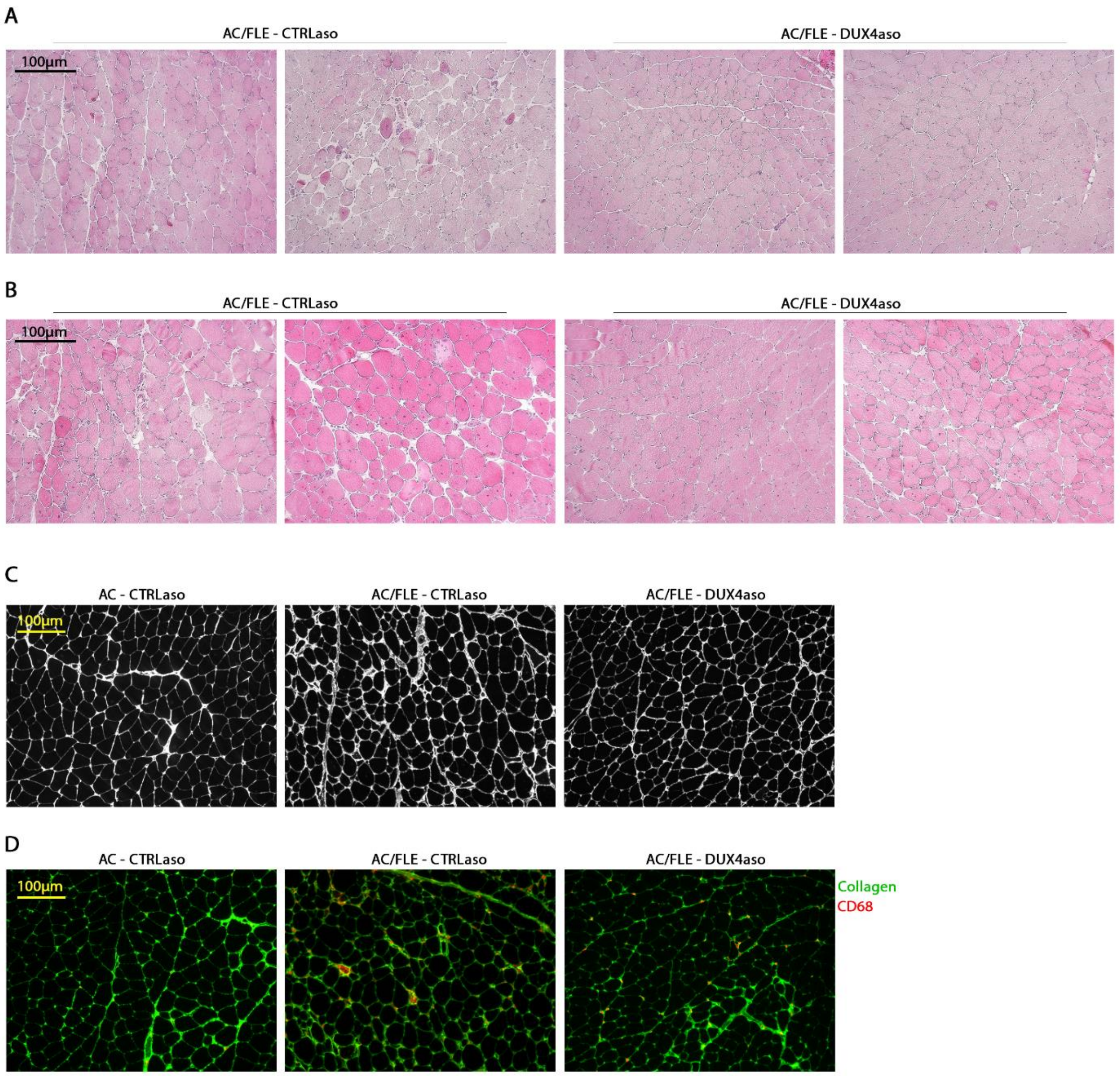

Figure S3: The DUX4 ASO reduced skeletal muscle pathology in ACTA1-MCM;FLExD mice. (A-B) Additional representative H&E stainings (100x magnification) of the quadriceps muscle (A) and the triceps muscle (B) of CTRLaso and DUX4aso treated ACTA1-MCM;FLExD mice of the long *in vivo* experiment. (C/D) Representative pictures of the collagen VI staining (C) and CD68 staining (D) on cryosections of the quadriceps muscle. A 100x magnification was used.

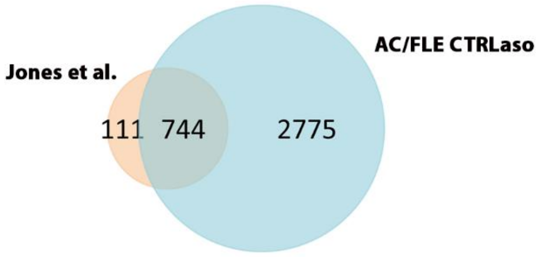

Figure S4: Overlap of differentially expressed genes compared to Jones et al.[27] Venn diagram representing the overlap between genes differentially expressed in the study of Jones et al. (ACTA1-MCM;FLExD versus ACTA1-MCM mice) and our analysis (CTRLaso treated ACTA1-MCM;FLExD versus CTRLaso treated ACTA1-MCM mice).

Supplementary file 1: List of genes up- or down-regulated (adjusted P-value <0.05) in the quadriceps muscle of ACTA1-MCM;FLExD and ACTA1-MCM mice receiving a long DUX4 ASO treatment. First tab: Differentially expressed genes in DUX4aso treated ACTA1-MCM;FLExD mice compared to CTRLaso treated ACTA1-MCM;FLExD mice. **Second tab:** Differentially expressed genes in CTRLaso treated ACTA1-MCM;FLExD mice compared to CTRLaso treated ACTA1-MCM mice. **Third tab:** Differentially expressed genes in DUX4aso treated ACTA1-MCM;FLExD mice compared to CTRLaso treated ACTA1-MCM mice. Padj = adjusted P-value.
